# Supplementary material for: Resolving the full spectrum of human genome variation using Linked-Reads
Source: Genome Res. 2019 Apr;29(4):635–45. doi: 10.1101/gr.234443.118 (PMC6442396; doi:10.1101/gr.234443.118)
Supplement: Supplemental Material [file supp_gr.234443.118_Supplemental_longranger-2.2.zip › longranger-2.2.tar.bz2!/lr2.2-public/lib/stan/cmdstan/stan/doc/api/html/agrad_2fwd_2functions_2owens__t_8hpp_source.html]

Stan: src/stan/agrad/fwd/functions/owens\_t.hpp Source File


|  |  |
| --- | --- |
|  | Stan  2.5.0  probability, sampling & optimization |


- Main Page
- Related Pages
- Namespaces
- Classes
- Files

- File List
- File Members

All Classes Namespaces Files Functions Variables Typedefs Enumerations Enumerator Friends Macros Pages

- src
- stan
- agrad
- fwd
- functions

owens\_t.hpp

Go to the documentation of this file.

1 #ifndef STAN\_\_AGRAD\_\_FWD\_\_FUNCTIONS\_\_OWENS\_T\_HPP

2 #define STAN\_\_AGRAD\_\_FWD\_\_FUNCTIONS\_\_OWENS\_T\_HPP

3

4 #include <math.h>

5 #include <stan/agrad/fwd/fvar.hpp>

6 #include <stan/meta/traits.hpp>

7 #include <stan/math/constants.hpp>

8 #include <stan/math/functions/owens\_t.hpp>

9 #include <stan/math/functions/square.hpp>

10

11 namespace stan {

12  namespace agrad {

13

14  template <typename T>

15  inline

16  fvar<T>

17  owens\_t(const fvar<T>& x1, const fvar<T>& x2) {

18  using stan::math::owens\_t;

19  using stan::math::pi;

20  using stan::math::INV\_SQRT\_2;

21  using stan::math::INV\_SQRT\_TWO\_PI;

22  using stan::math::square;

23  using std::exp;

24  using ::erf;

25

26  T neg\_x1\_sq\_div\_2 = -square(x1.val\_) \* 0.5;

27  T one\_p\_x2\_sq = 1.0 + square(x2.val\_);

28

29  return fvar<T>(owens\_t(x1.val\_, x2.val\_),

30  - x1.d\_

31  \* (erf(x2.val\_ \* x1.val\_ \* INV\_SQRT\_2)

32  \* exp(neg\_x1\_sq\_div\_2) \* INV\_SQRT\_TWO\_PI \* 0.5)

33  + x2.d\_ \* exp(neg\_x1\_sq\_div\_2 \* one\_p\_x2\_sq)

34  / (one\_p\_x2\_sq \* 2.0 \* pi()));

35  }

36

37  template <typename T>

38  inline

39  fvar<T>

40  owens\_t(const double x1, const fvar<T>& x2) {

41  using stan::math::owens\_t;

42  using stan::math::pi;

43  using stan::math::square;

44  using std::exp;

45

46  T neg\_x1\_sq\_div\_2 = -square(x1) \* 0.5;

47  T one\_p\_x2\_sq = 1.0 + square(x2.val\_);

48

49  return fvar<T>(owens\_t(x1, x2.val\_),

50  x2.d\_ \* exp(neg\_x1\_sq\_div\_2 \* one\_p\_x2\_sq)

51  / (one\_p\_x2\_sq \* 2.0 \* pi()));

52  }

53

54  template <typename T>

55  inline

56  fvar<T>

57  owens\_t(const fvar<T>& x1, const double x2) {

58  using stan::math::owens\_t;

59  using stan::math::pi;

60  using stan::math::square;

61  using stan::math::INV\_SQRT\_2;

62  using stan::math::INV\_SQRT\_TWO\_PI;

63  using std::exp;

64  using ::erf;

65

66  T neg\_x1\_sq\_div\_2 = -square(x1.val\_) \* 0.5;

67

68  return fvar<T>(owens\_t(x1.val\_, x2),

69  -x1.d\_ \* (erf(x2 \* x1.val\_ \* INV\_SQRT\_2)

70  \* exp(neg\_x1\_sq\_div\_2)

71  \* INV\_SQRT\_TWO\_PI \* 0.5));

72  }

73

74  }

75 }

76 #endif

stan::math::square

T square(const T x)

Return the square of the specified argument.

**Definition:** square.hpp:22

stan::math::INV\_SQRT\_TWO\_PI

const double INV\_SQRT\_TWO\_PI

**Definition:** constants.hpp:150

stan::agrad::erf

fvar< T > erf(const fvar< T > &x)

**Definition:** erf.hpp:17

stan::agrad::fvar

**Definition:** fvar.hpp:13

square.hpp

stan::agrad::square

fvar< T > square(const fvar< T > &x)

**Definition:** square.hpp:15

traits.hpp

stan::math::owens\_t

boost::math::tools::promote\_args< T1, T2 >::type owens\_t(const T1 &h, const T2 &a)

The Owen's T function of h and a.

**Definition:** owens\_t.hpp:64

stan::agrad::owens\_t

fvar< T > owens\_t(const fvar< T > &x1, const fvar< T > &x2)

**Definition:** owens\_t.hpp:17

stan::math::INV\_SQRT\_2

const double INV\_SQRT\_2

The value of 1 over the square root of 2, .

**Definition:** constants.hpp:26

fvar.hpp

owens\_t.hpp

stan::agrad::fvar::d\_

T d\_

**Definition:** fvar.hpp:16

stan::math::pi

double pi()

Return the value of pi.

**Definition:** constants.hpp:77

constants.hpp

stan::agrad::exp

fvar< T > exp(const fvar< T > &x)

**Definition:** exp.hpp:16

stan::agrad::fvar::val\_

T val\_

**Definition:** fvar.hpp:15


---

[ Stan Home Page ]

*© 2011–2014,
Stan Development Team.*
